# The role of robotic-assisted inguinal lymphadenectomy in vulval cancer: feasibility, peri-operative outcomes and oncological safety

**DOI:** 10.3389/fonc.2026.1892702

**Published:** 2026-08-17

**Authors:** Panagiwths Damianos, Theodoros Panoskaltsis, Stuart Rundle, Ali Kucukmetin, Porfyrios Korompelis

**Affiliations:** 1 2nd Department of Obstetrics and Gynaecology, Aretaieion University Hospital, Athens, Greece; 2 Gateshead Health NHS Foundation Trust, Gateshead, United Kingdom

**Keywords:** groin node dissection, inguinal node dissection, oncological outcome, post-operative outcome, robotic surgery, vulval cancer

## Abstract

Vulval cancer is a rare tumor, accounting for 3-5% of malignancies of the female reproductive system. The surgical management usually involves the radical excision of the tumor and inguinal node assessment for tumors with depth >1mm. Inguinal node dissection is usually performed as an open procedure through a 5-10cm incision in the groin area and carries a high risk of postoperative complications including wound infection, delayed wound healing, lymphocele and lymphedema which may result in prolonged hospitalization and delay in the adjuvant treatments for these patients. The role of minimal invasive surgery (MIS) in inguinal node dissection has been recently explored. Data extrapolated from melanoma and penile cancer patients showed that MIS inguinal node dissection is feasible, but it requires technical expertise and involves a steep learning curve. The aim of our study is to explore the feasibility and the oncological safety of the robotic inguinal node dissection for patients with vulval cancer, with secondary endpoint the intra- and post-operative outcomes for these patients compared to the open approach.

## Introduction

Vulvar cancer is a rare malignancy, accounting for 3–5% of cancers of the female reproductive tract (1). It represents approximately 0.5% of all cancer cases and 0.4% of cancer-related deaths among women worldwide (2). In Europe, vulvar cancer is the 19th most common malignancy among women (2). Although it predominantly affects older women, Kang et al. demonstrated an increasing incidence of vulvar cancer among women younger than 60 years in developed countries (3).

Squamous cell carcinoma (SCC) is the predominant histological subtype of vulvar cancer, accounting for 80–95% of all cases (4). Keratinizing SCC represents approximately 60% of vulvar SCCs and is primarily associated with chronic, non-infectious dermatoses in older women (4). In contrast, approximately 30% of SCCs are of the basaloid or warty subtype, which are associated with human papillomavirus (HPV) infection and typically occur in younger women (4).

Surgery remains the cornerstone of treatment for vulvar cancer and may be followed by adjuvant radiotherapy and/or chemotherapy when indicated. Radical local excision is the recommended surgical approach; however, the optimal depth of negative surgical margins remains a matter of debate (5). Inguinal lymph node assessment is indicated for tumors with a depth of invasion >1 mm. For patients with unifocal tumors measuring <4 cm and not clinically or radiologically suspicious inguinofemoral lymph nodes, sentinel lymph node (SLN) biopsy is recommended. A combination of a radioactive tracer (Tc-99m nanocolloid) with indocyanine green (ICG) and/or blue dye is commonly used for SLN identification (5). In contrast, patients with tumors >4 cm or multifocal disease should undergo complete inguinofemoral lymphadenectomy. Both the superficial and deep femoral lymph nodes should be removed, while preservation of the saphenous vein is recommended whenever feasible (5).

Groin node dissection is traditionally performed through a 5–10 cm transverse or vertical incision in the groin or upper medial thigh, with or without excision of a skin ellipse. In recent years, the role of minimally invasive surgery (MIS) for groin node dissection has been increasingly explored across several malignancies. In melanoma, Delman et al. were the first to describe a minimally invasive technique for inguinal lymphadenectomy (6). Subsequently, the three-port videoscopic approach was shown to achieve complication rates comparable to those of open surgery while reducing the severity of postoperative complications (7). Sommariva et al. further demonstrated that lymph node yield and regional recurrence rates were comparable between the minimally invasive and conventional open approaches (8).

Building on the experience gained in melanoma, Bishoff et al. reported the first laparoscopic inguinal lymph node dissection for penile cancer (9). Subsequently, Josephson et al. described the first robot-assisted endoscopic inguinal lymphadenectomy (10). These authors concluded that minimally invasive techniques provide satisfactory oncological outcomes while reducing surgical morbidity; however, they require considerable technical expertise and are associated with a steep learning curve (9).

The aim of the present review is to evaluate the feasibility and oncological safety of minimally invasive groin node dissection in patients with vulvar cancer, with particular emphasis on robot-assisted surgery. As a secondary objective, we compare the intraoperative and postoperative outcomes of robotic and conventional open approaches.

## Methodology

### Study design

This review was conducted to evaluate the feasibility, perioperative outcomes, and oncological safety of robotic groin node dissection in patients with vulvar cancer. It was designed as a narrative literature review with a structured search strategy to summarize the current evidence regarding minimally invasive groin node dissection in this patient population.

### Search strategy

A comprehensive literature search was performed in the PubMed/MEDLINE database between 1 October and 15 October 2025. The search strategy combined free-text keywords and Medical Subject Headings (MeSH) related to vulvar cancer and minimally invasive groin node dissection. The following search strategy was applied:

(“vulvar cancer” OR “vulvar carcinoma” OR “carcinoma vulva”) AND (“inguinofemoral lymphadenectomy” OR “inguinal lymphadenectomy” OR “inguinal lymph node dissection” OR “groin node dissection”) AND (“robotic” OR “robot-assisted” OR “laparoscopic” OR “endoscopic” OR “video-endoscopic” OR “minimally invasive”).

In addition, the reference lists of all eligible studies were manually screened to identify any relevant publications not retrieved through the primary database search.

### Study selection

A total of 47 records were identified through the PubMed search. Titles and abstracts were independently screened by two reviewers for relevance. Full-text articles considered potentially eligible were subsequently assessed according to predefined inclusion criteria. Additional studies identified through manual screening of reference lists were also evaluated for eligibility.

Studies were eligible for inclusion if they included adult patients with histologically confirmed vulvar carcinoma who underwent inguinofemoral or groin node dissection using a robot-assisted, laparoscopic, endoscopic, or other minimally invasive approach. Eligible studies were required to report perioperative and/or oncological outcomes, including operative time, estimated blood loss, lymph node yield, length of hospital stay, postoperative complications, recurrence, or survival outcomes.

**Figure 1:**
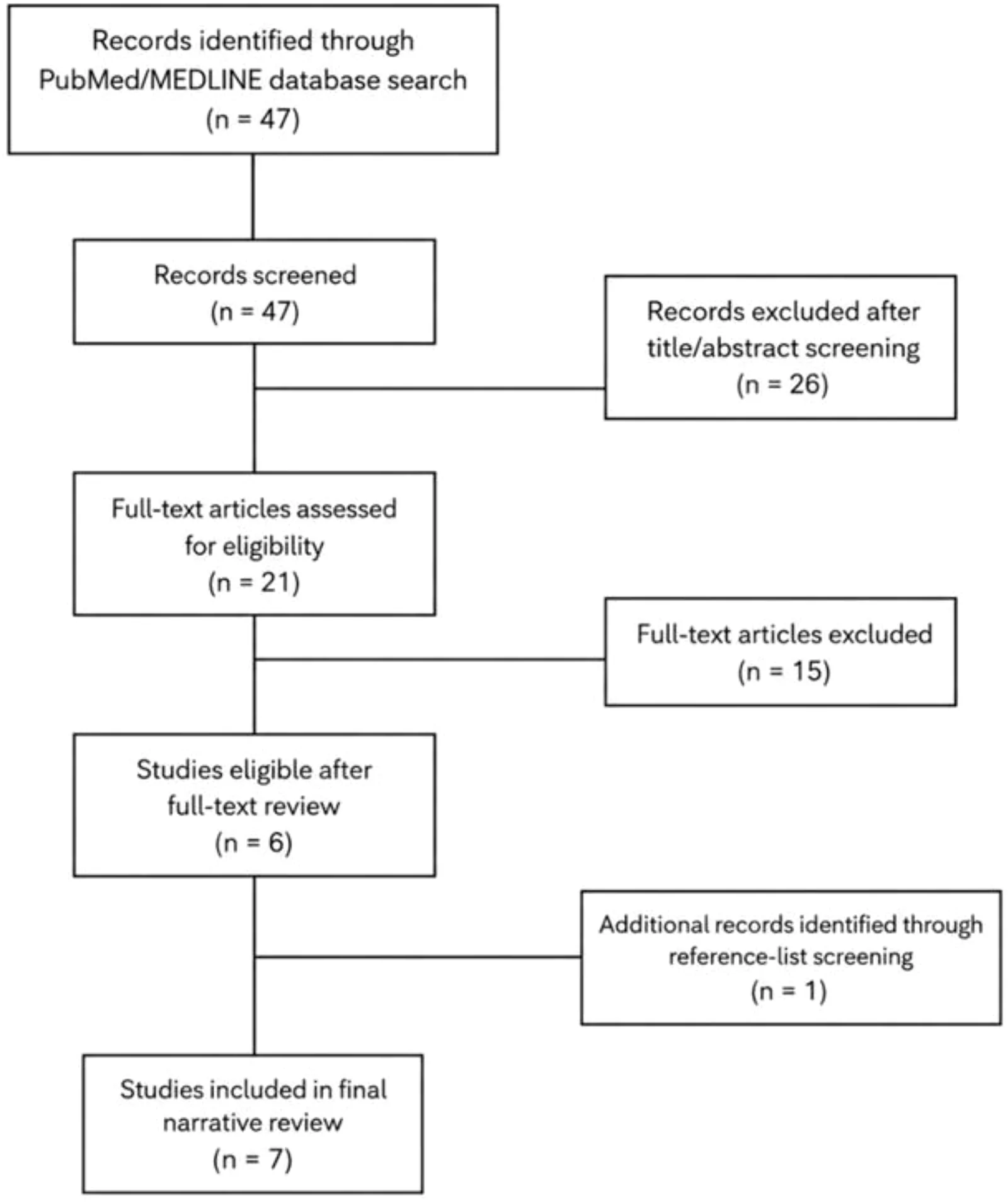
Flow chart of study selection.

Only English-language publications were included. Review articles, editorials, letters, and studies that did not report clinical outcomes relevant to robotic or minimally invasive groin node dissection in vulvar cancer were excluded. Following the screening and eligibility assessment process, seven studies were included in the final narrative synthesis (**Figure 1**).

### Data extraction

For each included study, data on authorship, year of publication, study design, country of origin, patient population, sample size, surgical technique, perioperative outcomes, and oncological outcomes were extracted. Particular attention was paid to operative time, estimated blood loss, length of hospital stay, lymph node yield, postoperative morbidity, recurrence rates, and duration of follow-up. Comparative outcomes between minimally invasive and conventional open approaches were recorded whenever available.

### Data synthesis

Given the limited number of available studies and the heterogeneity of study design, patient populations, surgical techniques, and reported outcomes, quantitative pooling of the results was considered inappropriate. Consequently, no formal statistical analysis or meta-analysis was performed. Instead, the findings were synthesized descriptively, with emphasis on the feasibility, safety, perioperative outcomes, and oncological adequacy of robotic groin node dissection.

### Limitations

This review has several limitations. As a narrative review, it is inherently susceptible to selection bias. Furthermore, the available literature consists predominantly of small retrospective case series, pilot studies, and case reports with limited follow-up. The small number of eligible studies and the heterogeneity of the reported outcomes precluded formal quantitative analysis. Nevertheless, this review provides a focused summary of the current evidence regarding robotic and other minimally invasive approaches to inguinofemoral lymphadenectomy in patients with vulvar cancer.

## Results

### Study selection

A total of seven studies met the eligibility criteria and were included in this review (**Table 1**). These comprised four small case series, one pilot feasibility study, one systematic review, and one comparative study. Sample sizes ranged from 4 to 27 patients, reflecting the rarity of minimally invasive groin node dissection in patients with vulvar cancer. Despite differences in study design, all included studies evaluated the technical feasibility, perioperative outcomes, and oncological safety of robotic and/or endoscopic approaches to groin node dissection.

**Table 1:** Summary of studies evaluating minimally invasive groin node dissection in vulvar cancer.

Study	Design/approach	Patients/groins	Operative time	Blood loss	Lymph node yield	Postoperative morbidity/recurrence	Follow-up
Zhang et al., 2017 (11)	Open ILND vs Video – endoscopic ILND	27 patients in the open group, 21 patients in the video-endoscopic group	Open 45,3 +/- 5.1 min Video – endoscopic ~109.9+/- 29.5 min	Open ~164.4 +/- 106.5 Video-endoscopic 214.8+/-204.4	Open 18+/-6 Video-endoscopic 15+/-5	Open 56% inguinal wound complications and 10% recurrence Video-endoscopic 5% inguinal wound complications and 10.5% recurrence	COIL group: 8–162 months, VEIL group:8–58 months
Jain et al., 2017 (12)	Robotic video-endoscopic ILND (R-VEIL)	12 patients, 22 groins	Mean 69.3 min (45–95)	Mean 30 mL	Mean 11 nodes (4–26)	Lymphocele, lymphedema, cellulitis; 1 recurrence	6–67 months
Mohammad et al., 2019 (13)	Robotic SLN mapping + ILND	4 patients, 8 groins	~234 min (with vulvar surgery)	~124 mL	Not reported	No major morbidity; no recurrences	3 years and it continues
Luan et al., 2021 (15)	VEIL (systematic review + single-center)	11 studies	33–180 min	9–96 mL	9–14 nodes	Lower wound morbidity; recurrence comparable	VEIL-L group: 1–67 months, Veil-H group: 3–162 months
Shah & Shah, 2025 (16)	Robotic vs open ILND	12 robotic vs 15 open	Robotic ~74.8 min; Open ~60.6 min	Not reported	Comparable	Fewer wound infections; similar recurrence	Median follow-up of 15 months,
Farghaly et al., 2015 (14)	Robotic vulvectomy + ILND	Small series	Not reported	Not reported	Not reported	No major intraoperative complications	Not reported
Iavazzo & Gkegkes, 2017 (17)	Review of robotic ILND	Review	—	—	—	Reduced wound morbidity; early oncologic safety	—

### Feasibility and operative outcomes

Robotic approaches were consistently reported to be technically feasible. The largest robotic series was published by Jain et al. (12), who performed 22 robot-assisted video-endoscopic inguinal lymphadenectomies in 12 patients. The mean operative time was 69.3 minutes, with minimal estimated blood loss (30 mL). No intraoperative complications were reported. While these results support feasibility, the study’s small numbers and lack of complication reporting raise concern about potential under-ascertainment of adverse effects, which is a common issue in early feasibility studies.

Mohammad et al. (13) reported similar findings in a small pilot series combining robotic sentinel lymph node mapping with inguinal lymphadenectomy. The reported operative time reflected the performance of concomitant vulvar procedures, limiting direct comparison with other studies. Mean estimated blood loss was 124 mL.

Farghaly et al. also reported successful completion of robotic vulvectomy with bilateral inguinofemoral lymphadenectomy. However, detailed perioperative outcomes were not provided, limiting comparison with other published series (DOI: 10.1016/j.jmig.2015.08.811) (14).

Overall, the available evidence suggests that robotic inguinal lymphadenectomy is technically feasible in carefully selected patients. However, the evidence is constrained by small sample sizes, non-standardized reporting and potential selection bias, since patients selected for the robotic surgery may have more favorable anatomical or disease characteristics.

Non-robotic minimally invasive techniques were reviewed by Luan et al. (15), who evaluated both the limb and hypogastric approaches to video-endoscopic inguinal lymphadenectomy. Reported operative times ranged from 33 to 180 minutes, likely reflecting differences in surgeon experience and patient selection, while the estimated blood loss remained consistently low across studies. These contextual modifiers may limit direct comparison among different studies but also suggest that minimally invasive approaches, irrespective of platform, can be performed safely with acceptable intraoperative outcomes.

### Lymph node yield

Jain et al. (12) reported a mean lymph node yield of 11 nodes per groin, comparable to that expected with conventional open surgery. In the systematic review by Luan et al. (15), lymph node yield ranged from 9 to 14 nodes, depending on the surgical approach. Similarly, Shah and Shah (16) reported no significant difference in lymph node retrieval between robotic and open procedures.

Although several smaller studies did not report detailed numerical data, all authors described adequate lymph node retrieval. Overall, minimally invasive approaches appear capable of achieving lymph node yields comparable to those of conventional open surgery, but the evidence is limited by small cohorts, non-uniform pathology protocols, and a lack of long-term oncologic correlation.

### Postoperative morbidity

Jain et al. (12) reported low rates of lymphocele formation and lower-limb lymphedema following robot-assisted video-endoscopic inguinal lymphadenectomy. Cellulitis and prolonged lymphorrhea were uncommon, and no intraoperative complications were reported. This absence of reported intraoperative complications may potentially reflect under-reporting rather than the true safety of the approach.

Similarly, Shah and Shah (16) observed fewer wound-related complications and earlier drain removal in the robotic group than in the open surgery group. Earlier drain removal is beneficial, but it may reflect institution-specific postoperative protocols rather than inherent advantages of the technique.

Mohammad et al. (13) likewise reported no cases of wound breakdown or persistent lymphedema following robotic surgery, however, the pilot nature of the study and short follow-up may limit conclusions about chronic morbidity.

The studies reviewed by Luan et al. (15) also demonstrated lower rates of wound dehiscence and skin necrosis following video-endoscopic inguinal lymphadenectomy than after conventional open surgery. Although lymphocele formation and prolonged lymphatic drainage were reported, their incidence was comparable to that observed following open inguinofemoral lymphadenectomy.

### Oncological outcomes

The available evidence suggests satisfactory short- to mid-term oncological outcomes following minimally invasive groin node dissection, although long-term follow-up remains limited. Jain et al. (12) reported one groin recurrence among twelve patients (8.3%) during follow-up.

Similarly, Shah and Shah (16) reported one recurrence among twelve patients (8.3%) in the robotic group and one recurrence among fifteen patients (6.7%) in the open surgery group after a median follow-up of approximately 15 months. No recurrences were reported in the pilot series by Mohammad et al. (13), although the small sample size limits interpretation.

In the systematic review by Luan et al. (15), recurrence rates were comparable between the two principal video-endoscopic approaches, with recurrence observed in 4.4% of patients undergoing the lower-extremity approach and 5.1% of those undergoing the lower-abdominal approach. Overall, the currently available – but very limited- evidence suggests that minimally invasive groin node dissection provides oncological outcomes comparable to those reported for conventional open surgery. However, larger prospective studies with longer follow-up are required to confirm these findings.

## Discussion

Vulvar cancer is a rare malignancy, accounting for 3–5% of cancers of the female reproductive tract. Standard surgical management consists of radical local excision combined with inguinal lymph node assessment for tumors with a depth of invasion >1 mm. Traditionally, groin node dissection is performed through an open approach using a 5–10 cm incision in the groin or upper medial thigh. Although oncologically effective, this procedure is associated with substantial postoperative morbidity.

The most common complications following open inguinal lymphadenectomy are wound-related, including wound infection, delayed wound healing, and skin necrosis, as well as lymphatic complications such as lymphocele formation and lower-limb lymphedema (18). These complications frequently prolong hospitalization and may delay the initiation of adjuvant treatment, thereby affecting both patient recovery and overall quality of care.

To reduce surgical morbidity, minimally invasive surgery (MIS) has increasingly been investigated for groin node dissection. Experience derived from melanoma and penile cancer has demonstrated that minimally invasive inguinal lymphadenectomy is technically feasible and associated with reduced postoperative morbidity, although it requires advanced technical expertise and involves a considerable learning curve (9). Furthermore, published studies suggest that minimally invasive approaches provide oncological outcomes comparable to those of conventional open surgery while reducing the severity of postoperative complications (7–9).

The present review evaluated the feasibility, perioperative outcomes, and oncological safety of robotic groin node dissection in patients with vulvar cancer. Although the published experience remains limited, the available evidence consistently supports the technical feasibility of the robotic approach. To our knowledge, based on the published literature identified in this review, fewer than 50 patients worldwide have undergone robot-assisted groin node dissection for vulvar cancer. The largest published series, reported by Jain et al. (12), included 22 robot-assisted video-endoscopic inguinal lymphadenectomies, demonstrating a mean operative time of 69.3 minutes, minimal estimated blood loss (30 mL), and no reported intraoperative complications. Although direct comparisons should be interpreted cautiously because of differences in study design and patient selection, these perioperative outcomes compare favorably with those reported for conventional open surgery.

One of the principal advantages of robotic surgery appears to be the reduction in postoperative morbidity. Large series of conventional open inguinal lymphadenectomy have reported wound breakdown in 9–24% of patients, cellulitis in 24–58%, groin infection in 3–27%, lymphocele formation in 12–27%, and chronic lymphedema in 21–34% (19–21). In contrast, the available robotic series consistently report lower rates of wound-related complications. Jain et al. observed low rates of lymphocele formation and lymphedema following robot-assisted surgery (12), while Shah and Shah reported fewer wound complications and earlier drain removal compared with the open approach (16). Similarly, Mohammad et al. described no cases of wound breakdown or persistent lymphedema following robotic inguinal lymphadenectomy (13). Although these findings are encouraging, they should be interpreted cautiously given the small study populations and limited follow-up.

From an oncological perspective, the currently available evidence is also reassuring. Lymph node yield, an important surrogate marker of surgical adequacy, appears comparable between robotic and conventional open surgery. Jain et al. reported a mean retrieval of 11 lymph nodes per groin (12), while Shah and Shah found no significant difference in lymph node yield between robotic and open procedures (16). Likewise, recurrence rates reported in the available studies remain low and appear similar between minimally invasive and conventional approaches. Nevertheless, these observations are based on small patient cohorts with relatively short follow-up, precluding definitive conclusions regarding long-term oncological safety.

Several limitations should be acknowledged when interpreting the findings of this review. First, the available evidence consists almost exclusively of small retrospective case series, pilot studies, and case reports, all of which are susceptible to selection and publication bias. Second, the limited number of reported patients, heterogeneous study designs, and variation in outcome reporting precluded quantitative synthesis of the available data. Finally, long-term oncological outcomes, including recurrence patterns and survival, remain insufficiently reported, limiting the strength of the current evidence.

An additional consideration is the cost and implementation of robotic surgery. Although no studies have directly compared the costs of robotic and open inguinal lymphadenectomy in patients with vulvar cancer, the economic sustainability of robotic programs generally depends on high procedural volumes. Given the rarity of vulvar cancer, this may limit the widespread adoption of robotic groin node dissection. Centralization of care within specialized referral centers may help overcome both economic challenges and the steep learning curve associated with these technically demanding procedures while allowing patients to benefit from advances in robotic technology, including emerging single-port platforms.

In conclusion, this review summarizes the current evidence regarding robotic and other minimally invasive approaches to inguinofemoral lymphadenectomy in patients with vulvar cancer. Robotic groin node dissection is a feasible alternative to conventional open surgery and appears to be associated with adequate lymph node retrieval, reduced postoperative morbidity, and encouraging short-term oncological outcomes. However, the available evidence remains limited, and larger prospective multicenter studies with longer follow-up are required before definitive conclusions can be drawn regarding its oncological equivalence and broader clinical adoption.
